# Flooding Length Mediates Fencing and Grazing Effects on Soil Respiration in Meadow Steppe

**DOI:** 10.3390/plants13050666

**Published:** 2024-02-28

**Authors:** Yan Qu, Deping Wang, Sanling Jin, Zhirong Zheng, Zhaoyan Diao, Yuping Rong

**Affiliations:** 1 College of Grassland Science and Technology, China Agricultural University, Beijing 100193, China; quyan162643@163.com (Y.Q.); shandongwangdeping@163.com (D.W.);; 2 State Environmental Protection Scientific Observation and Research Station for Hulunbuir Forest-Steppe Ecotone, Hulunbuir 021100, China; 3 State Environmental Protection Key Laboratory of Regional Eco-Process and Function Assessment, Chinese Research Academy of Environmental Sciences, Beijing 100012, China

**Keywords:** flooding condition, riparian grassland, heterotrophic respiration, autotrophic respiration, grazing, fencing

## Abstract

Grassland management affects soil respiration (Rs, consists of heterotrophic respiration and autotrophic respiration) through soil micro-ecological processes, such as hydrothermal, plant root, organic carbon decomposition and microbial activity. Flooding, an irregular phenomenon in grasslands, may strongly regulate the response of soil respiration and its components to grassland management, but the regulatory mechanism remains unclear. We conducted a 3-year experiment by grassland management (fencing and grazing) and flooding conditions (no flooding (NF), short-term flooding (STF) and long-term flooding (LTF)) to study their effects on Rs and its components in a meadow steppe in the Hui River basin of Hulunbuir. We found differences in the patterns of Rs and its components under grassland management and flooding conditions. In 2021–2023, the temporal trends of Rs, heterotrophic respiration (Rh) and autotrophic respiration (Ra) were generally consistent, with peaks occurring on days 190–220, and the peaks of grazing were higher than that of fencing. In NF, Rs of grazed grassland was significantly higher than that of fenced grassland in 2021–2022 (*p* < 0.05). In STF and LTF, there was no significant difference in Rs between fenced and grazed grassland (*p* > 0.05). The dependence of Rs on soil temperature (ST) decreased with increasing flooding duration, and the dependence of Rs on ST of grazed grassland was higher than fenced grassland under NF and STF, but there was no difference between fenced grassland and grazed grassland under LTF. In addition, Rh was more sensitive to ST than Ra. This may be due to the different pathways of ST effects on Rs under grazing in different flooding conditions. Our study indicates that the effect of flooding on Rs is the key to the rational use of grassland under future climate change. To reduce regional carbon emissions, we recommend grazing on flooding grassland and fencing on no-flooding grassland.

## 1. Introduction

The global annual flux of carbon through soil respiration (Rs) represents about 10% of the atmospheric carbon pool, is 10 times greater than the flux from fossil fuel combustion and is one of the largest sources of atmospheric CO_2_ [1]. Even small changes in Rs can have a significant impact on atmospheric CO_2_ concentrations, with further implications for climate change [2,3]. Soil respiration consists of two components: heterotrophic respiration (Rh), which is the CO_2_ released during microbial decomposition of organic matter, and autotrophic respiration (Ra), which includes CO_2_ released by live roots [4]. Identifying the different responses of Rs to human activities under global change is important for accurately predicting soil carbon storage under future climate change.

In grassland ecosystems, Rs can be severely disturbed by grazing through vegetation removal, manure deposition and trampling [5]. Many studies have investigated the response of Rs to grazed grasslands with mixed results, with some showing negative [6,7], neutral [8,9], or positive [10] effects of grazing. In general, grazing can reduce Rs by decreasing plant biomass and litter fall or increase Rs by enhancing the fertilizing effects of grazers’ urine and manure on plant growth and microbial activity [11,12]. The net effect of grazing on Rs should be attributed to the balance of these direct and indirect effects. In addition, factors associated with Rs are generally highly variable, and spatial heterogeneity exists within ecosystems [13,14]. Conflicting responses of Rs to grazing may depend on the spatial heterogeneity in grassland ecosystems [15,16]. Uncertainty in the Rs and its components for grassland management may affect the estimation of the total greenhouse gas budget in grassland ecosystems. Therefore, the spatial heterogeneity of environmental factors should be emphasized when studying the response of soil respiration and its components to grassland management.

Due to the presence of inland rivers, different flooding conditions lead to spatial heterogeneity of soil water distribution in grasslands. Therefore, flooding has a significant effect on ecological processes in grassland ecosystems [17]. Flooding generally promotes anaerobic conditions by excluding air from soil pore spaces, which reduces the mineralization of organic matter [18]. High concentrations of soil organic matter combined with warmer conditions in flooded soils can enhance CO_2_ production [19,20]. Several factors, such as the quality and quantity of substrates available for microbial processes, temperature, soil microbial activity and oxidation–reduction potential, can influence the magnitude of Rs [21,22]. This is likely to depend on the extent and duration of flooding [23]. The variation in flooding conditions might increase the uncertainty in soil respiration under grassland management, which would significantly hinder the full understanding of the global greenhouse gas budget. Further research is needed to fully understand the effects of flooding conditions under different grassland management on Rs and its components.

Considering previous studies of grassland management and flooding conditions on Rs and its components, we predicted that flooding would change grassland management effects on Rs and its components by regulating carbon availability and creating anaerobic conditions. The Hui River basin is located in the ecologically sensitive area of the Hulunbuir steppe, which is easily affected by climate change and human disturbance. Therefore, we conducted a continuous observation experiment to investigate the effects of grassland management (fencing and grazing) and flooding on soil respiration in the Hui River basin of the Hulunbuir steppe during the growing season, with the aim of answering the following questions: (a) how flooding affects the patterns of variability in Rs and its components under fencing and grazing and (b) how flooding regulates the dependence of Rs on soil temperature and water content and the pathways of Rs under fencing and grazing. By addressing these research questions, we hope to better understand how fencing and grazing affect soil respiration and its components under different flooding conditions in flooded areas. This knowledge can form the basis of management strategies for riparian grasslands and contribute to regional emission reductions.

## 2. Results

### 2.1. Temporal Variation in Rs and Its Components

**Figure 1:**
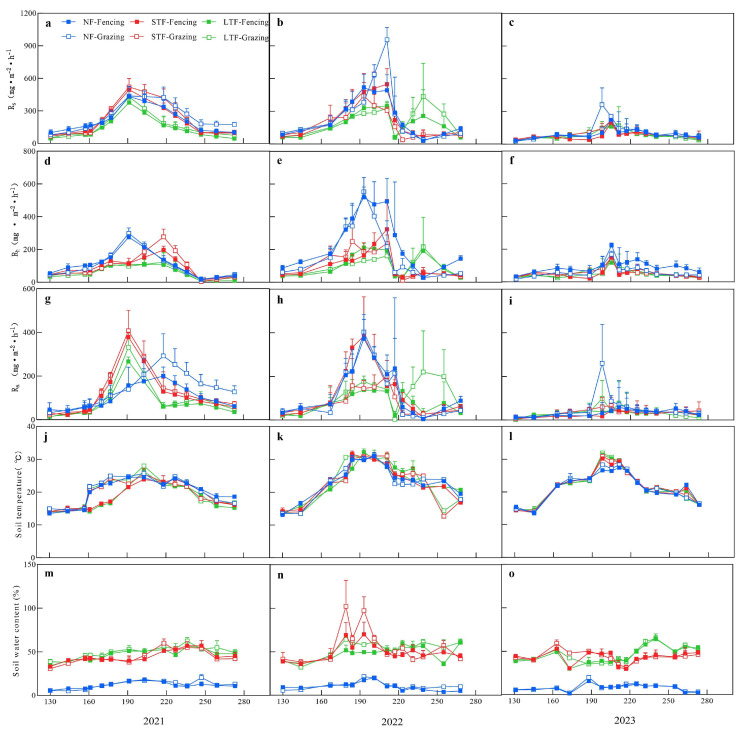
Temporal variation in soil respiration (Rs, (**a**–**c**)), heterotrophic respiration (Rh, (**d**–**f**)), autotrophic respiration (Ra, (**g**–**i**)), soil temperature (**j**–**l**) and soil water content (**m**–**o**) in different flooding conditions under fencing and grazing, respectively. NF, STF and LTF represent no flooding, short-term flooding and long-term flooding, respectively. Error bars represent standard errors of the mean (*n* = 4).

During the experiment, obvious temporal variations in soil temperature were observed from May 2021 to September 2023. Soil temperature showed similar temporal patterns among all treatments. Soil temperature increased from May to September and then gradually decreased, reaching a peak value on days 190–220 (Figure 1j–l). However, soil water content (SWC) fluctuated irregularly within a certain range and the range of SWC in NF, STF and LTF was 1.2–25%, 27.1–119.4% and 26.5–69.6%, respectively (Figure 1m–o). Rs and its components had similar temporal dynamics in all treatments and showed a trend of increasing and then decreasing over time, reaching the peak value on days 190–220 with higher soil temperature and soil water content (Figure 1j–o). Among them, the peaks of Rs, Rh and Ra appeared in 2021 in STF under grazing (520.4 mg·m^−2^·h^−1^), NF under grazing (298.3 mg·m^−2^·h^−1^) and STF under grazing (407.2 mg·m^−2^·h^−1^), respectively (Figure 1a,d,g). In 2022, the peaks of Rs (956.5 mg·m^−2^·h^−1^), Rh (403.6 mg·m^−2^·h^−1^) and Ra (298.3 mg·m^−2^·h^−1^) all occurred in NF under grazing (Figure 1b,e,h). In 2023, the peaks of Rs (255.2 mg·m^−2^·h^−1^), Rh (168.7 mg·m^−2^·h^−1^) and Ra (70.0 mg·m^−2^·h^−1^) all occurred in NF under grazing (Figure 1c,f,i).

### 2.2. Effects of Grassland Management and Flooding Conditions on Rs and Its Components

**Figure 2:**
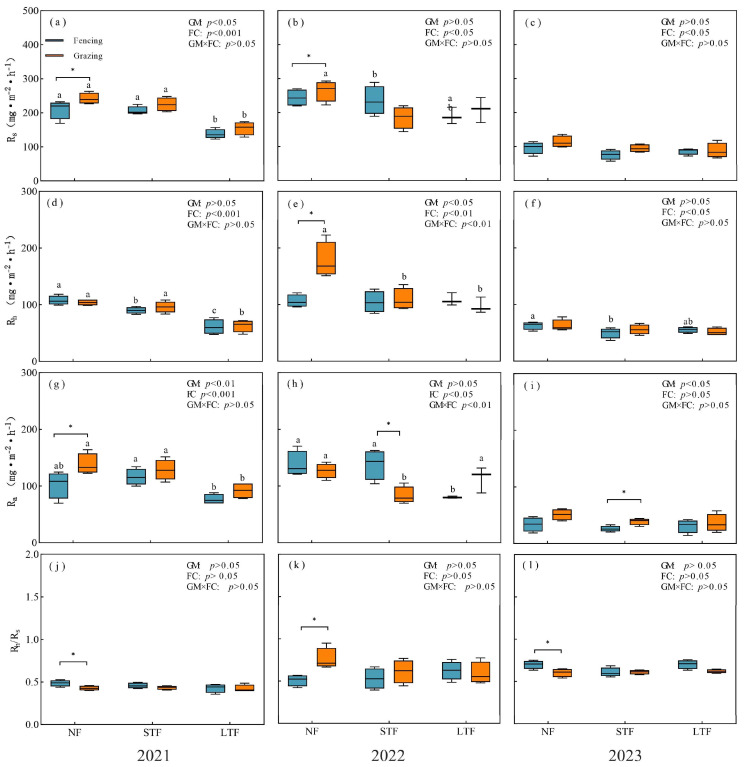
Responses of soil respiration (Rs, (**a**–**d**)), heterotrophic respiration (Rh, (**e**–**h**)) and autotrophic respiration (Ra, (**i**–**l**)) to different flooding conditions under fencing and grazing. Mean ± standard error (SE). Different letters indicate significant differences between flooding conditions (*p* < 0.05). * indicate significant differences between fencing and grazing at *p* < 0.05. GM and FC represent grassland management and flooding conditions, respectively. NF, STF and LTF represent no flooding, short-term flooding and long-term flooding, respectively.

Year, grassland management and flooding conditions had significant effects on Rs and its components (Table S1). In 2021, 2022 and 2023, grazing increased Rs by 15.0%, 8.4% and 17.8% in NF, respectively (Figure 2a–c). In 2021, Ra of grazed grassland was significantly higher than that of fenced grassland (*p* < 0.05, Figure 2g), and Rh had no significant difference (*p* > 0.05, Figure 2d). In 2022, Rh of grazing grassland was significantly higher than that of fencing grassland (Figure 2e), and Ra had no significant difference (Figure 2h). In 2023, there was no significant difference in Rh and Ra between grazing and fencing (Figure 2f,i). Grazing significantly reduced Ra in 2022 and increased it in 2023 in STF (Figure 2d–i). Compared with NF, LTF reduced Rs, Rh and Ra in grazed grassland but only showed significant differences in 2021. In all treatments, the Rh/Rs ratio is greater than 0.5. In NF, grazing significantly reduced the Rh/Rs ratio in 2021 and 2023 but significantly increased the Rh/Rs ratio in 2022. However, there was no significant difference in the Rh/Rs ratio between fenced grassland and grazed grassland in STF and LTF in 2021, 2022 and 2023 (Figure 2j–l).

### 2.3. Relationship of Rs and Its Components with Soil Temperature and Water Content

**Figure 3:**
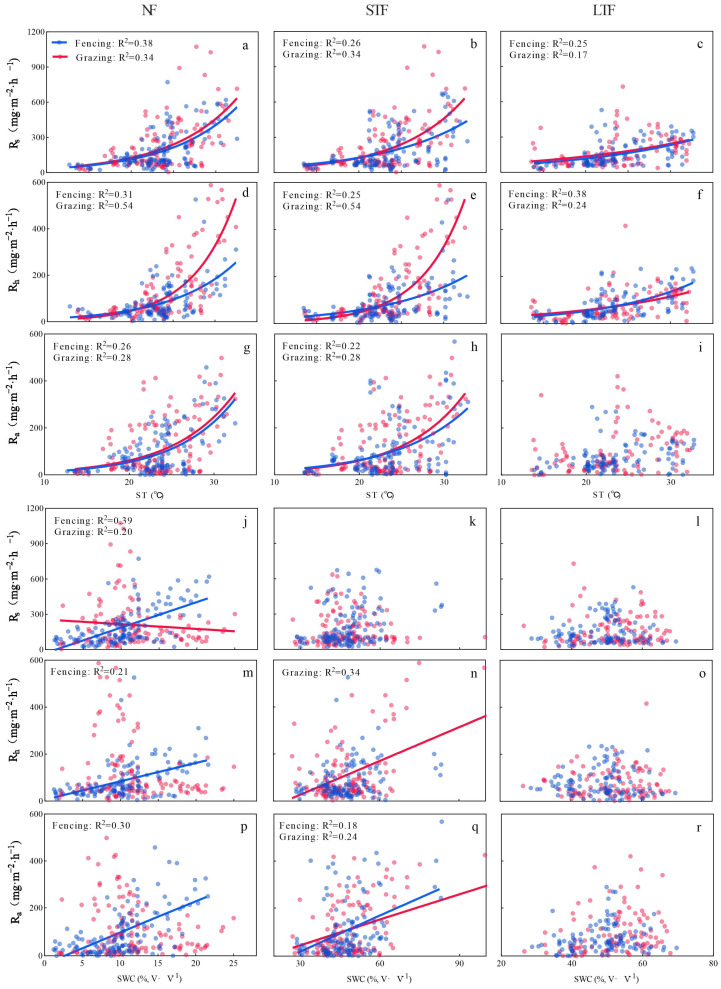
Relationships of soil respiration (Rs), heterotrophic respiration (Rh), autotrophic respiration (Ra) with soil temperature (ST, (**a**–**i**)) and soil water content (SWC, (**j**–**r**)) under different grassland management and flooding conditions during the growing seasons from 2021 to 2023. NF, STF and LTF represent no flooding, short-term flooding and long-term flooding, respectively.

In all treatments, soil temperature (ST) had an exponential relationship with Rs and its components, with R^2^ ranging from 0.17 to 0.54 (Tables S2 and S3). The rate of increase of Rs and its components with ST under grazing was higher than that under fencing in NF and STF. However, the rate of increase of Rs and its components with ST was similar under grazing and fencing in LTF. Under NF, the rate of increase of Ra (the increase rate was 0.14) with ST was higher than Rs (the increase rate was 0.13) and Rh (the increase rate was 0.13) in fenced grassland, while the rate of increase of Rh (the increase rate was 0.19) with ST was higher than Rs (the increase rate was 0.13) and Ra (the increase rate was 0.14) in grazed grassland (Figure 3a,d,g and Table S2). Under STF and LTF, the rate of increase of Rh with ST was higher in fenced and grazed grassland than that of Rs and Ra (Figure 3b,c,e,f,h,i and Table S2). In NF, soil water content (SWC) was positively related to Rs and its components in fenced grassland but negatively related in grazed grassland (Figure 3j,m,p). In STF, SWC was positively related to Rh and Ra in grazing grassland and to Ra in fenced grassland (Figure 3k,n,q). In LTF, SWC had no significant relationship with Rs and its components in grazing and fenced grassland (Figure 3l,o,r).

### 2.4. Direct and Indirect Pathways of Rs and Its Components

**Figure 4:**
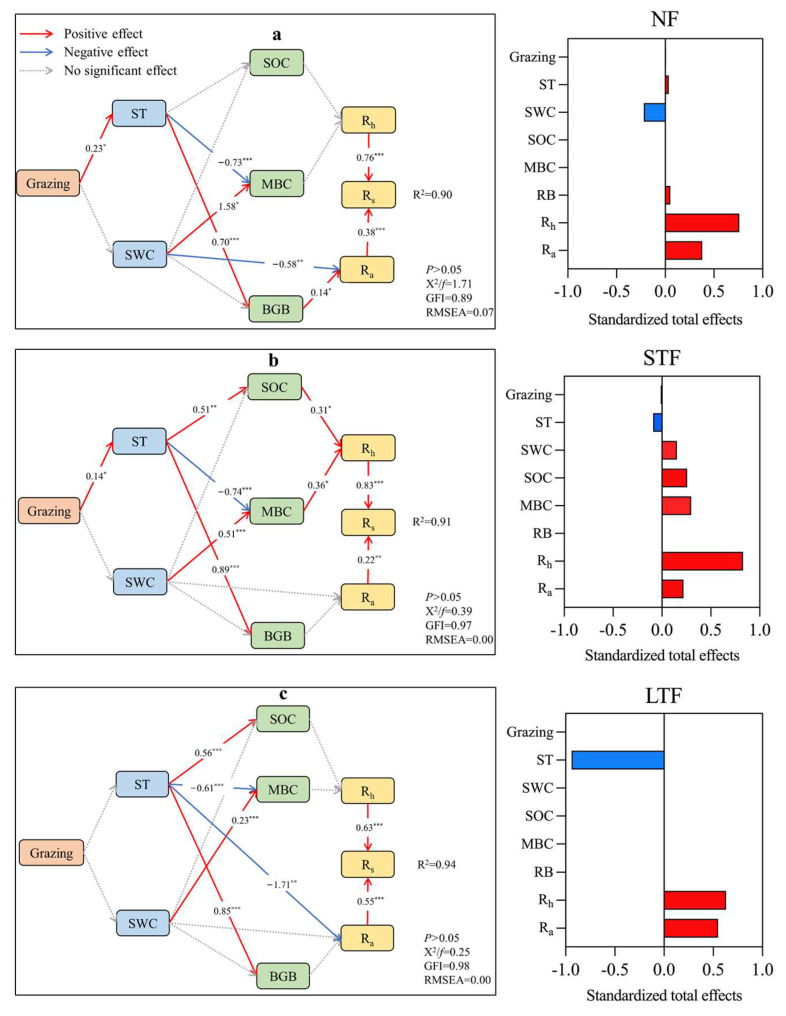
Partial least squares path modeling (PLS-PM) of grassland management and flooding conditions on soil respiration (Rs), heterotrophic respiration (Rh) and autotrophic respiration (Ra). *, ** and *** indicate significance at the 0.05, 0.01 and 0.001 levels. FC, flooding condition; ST, soil temperature; SWC, soil water content; SOC, soil organic carbon; MBC, microbial biomass carbon; BGB, belowground biomass.

Based on the results of the hierarchical partitioning (Table S5), the dominant factors that drove the variances in Rs and its components were applied in partial least squares path modeling (PLS-PM) to illustrate direct and indirect paths of controlling factors to Rs and its components (Figure 4). The PLS-PM explained 90%, 91% and 94% of the variance in Rs of the no flooding (NF), short-term flooding (STF) and long-term flooding (LTF) grassland, respectively. The results showed that Rs was directly regulated by Rh and Ra and that Rh had a greater effect on Rs than Ra. In NF, belowground biomass (BGB) was the main predictor of Ra (0.14 of the direct effect), with grazing increasing BGB by positively affecting ST (0.70 of the direct effect, Figure 4a). In STF, grazing increased ST (0.14 of the direct effect), which in turn reduced microbial biomass carbon (MBC) (−0.74 of the direct effect), while MBC increased Rh (0.36 of the direct effect) by positively affecting Rs (0.83 of the direct effect, Figure 4b). In LTF, grazing did not regulate Rs by affecting ST and SWC (Figure 4c).

## 3. Discussion

We found that Rs and its components (Rh and Ra) showed a similar trend under different grassland management (fencing and grazing) during the growing seasons in 2021, 2022 and 2023, which showed a close relationship with soil temperature and soil water content (Figure 2). Soil temperature (ST) and soil water content (SWC) jointly explained more than 50% of the variance (Table S4) [24]. Soil respiration increased with increasing ST during the growing season with the identification of one peak in all treatments (Figure 1). A similar trend of Rs was also observed in some studies conducted in floodplain forests of the Danube National Park [25,26]. The peak of Rs in the wet meadow occurred around early August, which generally coincided with the peak of soil temperature at 5 cm depth. Importantly, the dependence of Rs on ST under grazed grasslands was higher than that of fenced grasslands, indicating that grazed grasslands have a higher potential for soil CO_2_ emission under future climate warming. Furthermore, there was a linear relationship between Rs and SWC (Figure 3j). This suggests that precipitation may influence the temporal patterns of Rs. Higher precipitation resulted in higher SWC, poorer aeration conditions and limited gas diffusion, which are unfavorable for Rs. When precipitation decreased, CO_2_ was released from the soil to the atmosphere. The different responses of Rs to ST and SWC may be largely due to the different effects of the two components of Rs. It is worth noting that ST and SWC affected Rs both individually and additively. In summary, it can be concluded that the annual variation of soil respiration is mainly influenced by soil temperature, while the inter-annual variation is caused by precipitation. In order to better explain the temporal variation in soil respiration and its components, it is necessary to pay attention to changes in soil temperature and water.

The effects of grassland management on Rs and its components are regulated by a variety of factors [27,28,29]. We emphasized that the effects of fencing and grazing on Rs and its components were mediated by flooding conditions. In the no flooding (NF) plot, grazing significantly increased Rs, with changes mainly driven by Ra. Autotrophic respiration was mainly driven by root respiration, while grazing led to an increase in belowground biomass (BGB) by increasing ST (Figure 3c). In the short-term flooding (STF) and long-term flooding (LTF) plots, there was no significant difference in Rs between fenced and grazed grassland. In the STF plot, the change in Rs was driven primarily by Rh. Heterotrophic respiration was driven primarily by SOC and MBC simultaneously. Grazing increased SOC by increasing soil temperature while decreasing MBC. Overall, the effect of soil temperature on MBC and SOC was offset. Through comprehensive analysis, Xu et al. [30] found that grazing could significantly reduce MBC, which may be because grazing stimulates the reallocation of plant biomass towards belowground, and this promotion of root growth helps to maintain soil moisture, and therefore, soil microorganisms thrive [31,32]. Many studies have suggested that grazing reduced SOC, which may be due to the fact that the study site was in arid grassland [33,34]. However, anaerobiosis is thought to reduce decomposition rates compared to aerobic conditions [35]. Activities of soil hydrolytic enzymes, which directly control soil organic matter decomposition, are thought to decrease under anaerobic conditions due to reduced enzyme production and inhibition by phenolic substances [36]. In addition, reactive soil minerals, particularly iron (Fe) phases, play a critical role in protecting soil carbon from microbial decomposition. For example, hydrophilic and carboxylic carbon, which are readily assimilated by microbes, can be stabilized by Fe oxides via sorption and co-precipitation [37]. In particular, protective associations between Fe mineral phases and SOC may be vulnerable to moisture-sensitive redox dynamics [38,39,40]. In contrast, there were no direct or indirect effects on SOC and MBC in the LTF plot. Flooding conditions significantly affected Rs, possibly by altering aerobic/anaerobic conditions, redox potential, soil water content, soil organic carbon and microbial activities [41]. In our study, flooding significantly reduced Rs and Rh under STF and LTF compared with NF. The possible explanation is that flooding not only inhibited CO_2_ diffusion from soil to air but also reduced microbial decomposition activity and ecosystem respiration [42,43]. Similar results were reported in a comprehensive analysis involving several different grassland ecosystems, where grazing-induced reductions in soil greenhouse gas fluxes occurred in dry grasslands with precipitation below 400 mm, but not in relatively wet grasslands with precipitation above 400 mm [44]. Nevertheless, grazing could only significantly improve Rh in NF. This implies that soil carbon cycling and belowground biotic processes are more vulnerable to grazing pressure in dry grasslands than in wet grasslands due to low plant productivity, soil water and nutrient availability [45]. The possible explanation is that, as with STF and LTF, low SOC concentrations and substrate availability in soil layers may limit soil respiration [46]. The PLS-PM analysis showed that flooding did not affect the control pathway of Rs by fencing and grazing. This is because flooding masks the effects of grassland management on Rs. In addition, although flooding could increase Rh via its positive effect on MBC, this positive effect was outweighed by the negative effect of flooding on SOC. These results are also supported by studies conducted in a rice field in Japan [47] and a peatland ecosystem [48,49] and a coastal wetland in the Yellow River Delta, China [50]. Our results suggest that grazing does not necessarily increase ecosystem carbon loss, as flooding has a significant influence on this process. Therefore, quantifying the response of Rs and its components to global change has important implications for better predicting future ecosystem carbon cycling and its feedback to the climate system in ecosystem models.

## 4. Materials and Methods

### 4.1. Study Site

**Figure 5:**
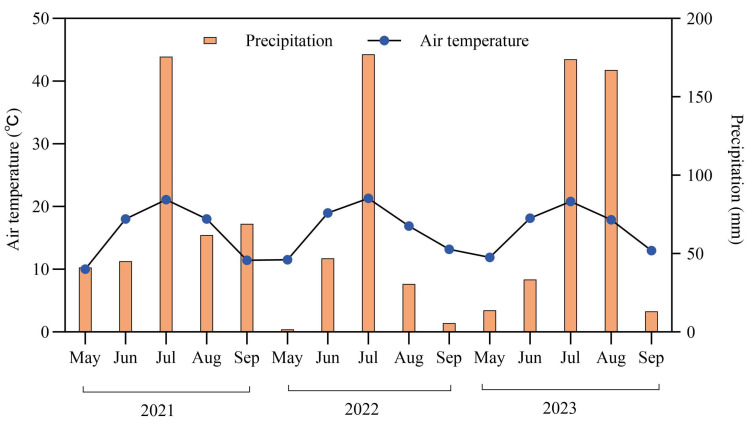
Temporal variations in air temperature and precipitation from 2021 to 2023 in the study area.

The field experiment was conducted in the Hui River National Nature Reserve, Hulunbuir, Northeast China (47°36–49°01′ N, 118°48–120°21′ E, 800–1000 m). The region is strongly influenced by the continental monsoon climate. The average air temperature during the growing season (June and September) was 1.34 °C. The frost-free period was between 100 and 120 days, and the average annual precipitation was 395.1 mm from 2021 to 2023 (Figure 5). The vegetation is classified as temperate meadow steppe, dominated by *Leymus chinensis*, *Stipa baicalensis*, *Artemisia tanacetifolia*, *Carex duriuscula*, *Cleistogenes squarrosa* and *Potentilla acaulis*.

### 4.2. Experimental Design

To investigate the effects of grassland management and flooding conditions on soil respiration and its components, a split-plot design experiment was set up in May 2021. The main block was grassland management (fencing and grazing) and the sub-block was flooding conditions, which were no-flooding (NF), short-term flooding (STF, flooding time less than 1 month per year) and long-term flooding (LTF, flooding time more than 3 months per year). Each treatment was replicated four times, for a total of 24 plots. The area of each plot was 10 m × 30 m. The fencing treatment has involved no grazing since 2008. Grazing was carried out at a stocking rate of 6.14 sheep ha^−1^ from May to September. The grazing mode of each plot was free grazing.

### 4.3. Measurements of Rs and Its Components

In 2021, two types of polyvinyl chloride (PVC) collars (20 cm internal diameter) were installed in the ground in each plot. Rs was measured using PVC collars (15 cm high) inserted 10 cm into the soil, and Rh was measured using PVC collars (35 cm high) inserted 30 cm into the soil. The depth of 30 cm was chosen in order to remove most of the living plant roots, as fine roots were mainly present in the top 30 cm of the soil profile at the experimental site. Ra was calculated by the formula Rs minus Rh [51,52].

Rs was measured using a static opaque chamber and gas chromatography [53,54]. The chambers (30 cm high and 20 cm external diameter) were sealed at the top and fitted with a hose with a valve to connect to the inside of the chamber. A fan was installed in each chamber to mix the air during sampling, and a thermometer was placed in the chamber to measure the atmospheric temperature. Aboveground biomass was removed from the PVC collar approximately 24 h before each measurement period. Gas samples were taken from the chamber with plastic syringes at 10 min intervals over a 30 min period. Carbon dioxide (CO_2_) concentrations in the gas samples were analyzed using a greenhouse gas concentration analyzer (G2308; Picarro; Beijing, China). CO_2_ concentrations were calculated using a linear least squares fit to the four points in the time series of gas concentration in each chamber with an average chamber temperature. Data were omitted if the coefficient of determination (R) of the slope of the linear fit was <0.90. Soil respiration was calculated using the following formula:

$$F=\frac{d_{c}}{d_{t}}\;\times \;\frac{M}{V_{0}}\;\times \;\frac{P}{P_{0}}\times \;\frac{T_{0}}{T}\times \;H$$

where *F* is soil respiration (mg·m^−2^·h^−1^), *c* is the gas concentration (μg·m^−3^), and *d_c_/d_t_* is the slope of the straight line when the gas concentration changes with the sampling time. *M* is the molar mass of the measured gas. *P* is the sampling point pressure. *t* is the absolute temperature of gas collection. *V*_0_, *P*_0_ and *T*_0_ are volume, air absolute temperature and air pressure in standard state, respectively. *H* is the height of the sampling chamber [55].

From the beginning of May 2021 to the end of September 2023, all treatments were sampled for gas two to four times per month between 8:00 and 11:30 on sunny days. A total of 43 measurements per treatment were taken during the measurement period. Soil temperature (ST, °C) and soil water content (SWC, V·V^−1^) were measured at a depth of 5 cm in each plot using a portable soil moisture temperature conductivity meter (POGO mini; STVNS; Beijing, China). All measurements were taken simultaneously with soil respiration measurements.

### 4.4. Aboveground and Belowground Biomass Measurements

Plant samples were collected in mid-August from 2021 to 2023 in each plot. Aboveground plant samples were clipped from two 1 m × 1 m sampling frames in each plot. Belowground samples were collected from a depth of 10 cm using a 7 cm diameter soil sampler (QTZ; Sanyu; Zhejiang, China), and five subsamples were collected from each plot and separated from the soil by washing. All plant samples were dried at 65 °C and weighed to calculate aboveground and belowground biomass.

### 4.5. Soil Sampling and Analysis

In late August 2021, 2022 and 2023, five soil cores (5 cm in diameter and 10 cm in depth) were collected from each plot and pooled to form a composite soil sample. Composite soil samples for each plot were sieved (2 mm mesh) to remove organic matter and stones. A portion of the fresh soil sample was stored at −20 °C and analyzed for soil microbial carbon (MBC), soil microbial nitrogen (MBN), ammonium (NH_4_^+^-N) and nitrate (NO_3_^−^-N). The other portion of fresh soil was air-dried in a shaded and ventilated environment until a constant weight was reached for other soil physiochemical analyses.

Soil total carbon (TC) and nitrogen (TN) were analyzed using an elemental analyzer (Vario EL III; Elementar; Hanau, Germany). Soil organic carbon (SOC) was analyzed by external heating with potassium dichromate. Concentrations of NH_4_^+^-N and NO_3_^−^-N were analyzed using 50 mL of 2 mol/L KCl solution and a colorimetric analysis with filtered KCl extractant using an automatic chemical analyzer (EasyChem plus; Systea; Anagni, Italy). Soil pH of the air-dried soil was measured using a pH meter (model PHS-2; INESA Instrument; Shanghai, China) with a soil–water ratio of 1:5. Soil microbial biomass carbon (MBC) and nitrogen (MBN) were analyzed using the chloroform fumigation-K_2_SO_4_ extraction method with a TOC analyzer (Multi N/C 3100; Analytikjena; Jena, Germany) [56].

### 4.6. Data Analysis

Repeated measures analysis of variance (ANOVA) was used to investigate the effects of year, grassland management, flooding condition and their interactions on Rs, Rh and Ra. The year was used as a repeated factor and flooding condition and grassland management as treatment factors. The relationship between soil respiration and soil temperature (ST) was fitted by the following exponential equation (Rs = a × exp(b × ST)). Linear regression analysis was used to determine the relationships between soil water content (SWC) and Rs and its components. Stepwise multiple regression analysis was used to evaluate the influence of soil temperature and soil water content on Rs and its components. Hierarchical partitioning (rdacca.hp function package in R) was used to rank the importance of the main factors influencing Rs and its components. Based on the results of hierarchical partitioning, the dominant factors were incorporated into partial least squares path modeling (PLS-PM) to illustrate direct and indirect pathways of Rs and its components. All statistical analyses were performed using SPSS statistical software (SPSS 22.0 for Windows; SPSS Inc., Chicago, IL, USA). And we use GraphPad Prism 10 for graphic visualization.

## 5. Conclusions

In the Huihe River Basin, fenced and grazed grasslands in no-flooding, short-term flooding and long-term flooding were selected as research objects to compare the changes in soil respiration and its influencing processes. The results showed that soil respiration (Rs), heterotrophic respiration (Rh) and autotrophic respiration (Ra) had similar temporal variations, which peaked at 190–220 days in 2021–2023, with a higher peak in grazing than in fencing. Importantly, our results highlight the importance of flooding conditions for Rs and its components in fenced and grazed grassland. Flooding reduced differences in Rs and their dependence on ST under fenced and grazed grassland, and the dependence of Rs on ST decreased with increasing duration of flooding. It is noteworthy that Rh contributed more to Rs than Ra and was more sensitive to ST than Ra. This might be due to the different pathways by which ST affected Rs under grazing under different flooding conditions. These results improve the understanding of the mechanisms underlying Rs and its components, which is necessary for regional carbon emission reduction and riparian grasslands’ sustainable use. Therefore, grazing on flooding grasslands and fencing on no-flooding grasslands should be considered when developing riparian grassland management strategies.

## Data Availability

Data are contained within the article and supplementary materials.

## Supplementary Materials

The following supporting information can be downloaded at https://www.mdpi.com/article/10.3390/plants13050666/s1, Table S1: Results of three-way ANOVAs showing the effects of grassland management, flooding conditions, years and their interaction on Rs and its components; Table S2: Exponential equation of soil respiration (Rs), heterotrophic respiration (Rh) and autotrophic respiration (Ra) with soil temperature. Table S3: Regression equation of soil respiration (Rs), heterotrophic respiration (Rh) and autotrophic respiration (Ra) with soil water content. Table S4: Linear regression equations between soil respiration (Rs), heterotrophic respiration (Rh), autotrophic respiration (Ra) and soil temperature and soil water content. Table S5: Influencing factors of Rs and its components under different flooding conditions
