# Supplementary material for: Flooding Length Mediates Fencing and Grazing Effects on Soil Respiration in Meadow Steppe
Source: Plants (Basel). 2024 Feb 28;13(5):666. doi: 10.3390/plants13050666 (PMC10934994; doi:10.3390/plants13050666)
Supplement: Supplementary file 1 [file plants-13-00666-s001.zip › plants-2851087-supplementary.pdf]

Table S1 Results of three-way ANOVAs showing the effects of grassland management, flooding conditions, years and their interaction on Rs and its components.

	GM	FC	Year	GM×FC	GM×Year	FC×Year	GM×FC×Year
Rs	3.15	23.35***	163.75***	2.11	1.33	5.76**	2.04
Rh	8.36**	28.76***	112.89***	4.55*	3.64*	5.30**	8.02***
Ra	1.75	13.22***	146.01***	4.29*	6.43**	4.67**	5.73**
Rh/Rs	0.06	1.45	39.79***	1.13	5.98**	0.47	2.91*

*, ** and *** represents the statistical significance was at the level of $p < 0.05$, 0.01 and 0.001, respectively. GM, grassland management; FC, flooding condition; Rs, soil respiration; Rh, heterotrophic respiration; Ra, autotrophic respiration.

Table S2 Exponential equation of soil respiration (Rs), heterotrophic respiration (Rh) and autotrophic respiration (Ra) with soil temperature

Treatment		Equation	N	*p*	R^2
		Rs=8.95×exp(0.13×ST)	172	<0.001	0.38
	NF	Rh=3.62×exp(0.13×ST)	172	<0.001	0.31
		Ra=3.16×exp(0.14×ST)	172	<0.001	0.26
		Rs=17.88×exp(0.10×ST)	172	<0.001	0.26
Fencing	STF	Rh=0.10×exp(7.70×ST)	172	<0.001	0.25
		Ra=5.88×exp(0.12×ST)	-	-	0.22
		Rs=29.50×exp(0.07×ST)	172	<0.001	0.25
	LTF	Rh=8.27×exp(0.09×ST)	172	<0.001	0.38
		Ra=25.22×exp(0.04×ST)	172	<0.001	＜0.20
		Rs=9.50×exp(0.13×ST)	172	<0.001	0.34
	NF	Rh=1.00×exp(0.19×ST)	-	-	0.54
		Ra=3.79×exp(0.14×ST)	-	-	0.28
		Rs=37.86×exp(0.06×ST)	172	<0.001	＜0.20
Grazing	STF	Rh=11.83×exp(0.09×ST)	172	<0.001	0.24
		Ra=27.58×exp(0.05×ST)	172	<0.001	＜0.20
		Rs=42.01×exp(0.06×ST)	172	<0.001	＜0.20
	LTF	Rh=14.27×exp(0.07×ST)	172	<0.001	0.22
		Ra=27.04×exp(0.05×ST)	172	<0.001	＜0.20

Table S3 Regression equation of soil respiration (Rs), heterotrophic respiration (Rh) and autotrophic respiration (Ra) with soil water content

Treatment		Equation	N	*p*	R^2
Fencing	NF	Rs=21.72×SWC−29.62	172	<0.001	0.4

		Rh=7.82×SWC+6.23	172	<0.001	0.21
		Ra=13.13×SWC−34.49	172	<0.001	0.3
		Rs=5.52×SWC−71.69	172	<0.001	＜0.20
	STF	Rh=2.06×SWC−10.69	172	<0.001	＜0.20
		Ra=4.99×SWC−130.90	-	-	＜0.20
		Rs=1.05×SWC+104.30	-	-	＜0.20
	LTF	Rh=−0.02×SWC+81.65	-	-	＜0.20
		Ra=1.03×SWC+24.12	-	-	＜0.20
		Rs=−4.02×SWC+256.30	-	-	＜0.20
	NF	Rh=13.25×SWC−28.34	-	-	0.23
		Ra=13.37×SWC−37.43	-	-	0.3
		Rs=−0.76×SWC+199.80	-	-	＜0.20
Grazing	STF	Rh=1.80×SWC+3.83	172	<0.001	＜0.20
		Ra=0.57×SWC+59.61	-	-	＜0.20
		Rs=0.94×SWC+108.1	-	-	＜0.20
	LTF	Rh=0.48×SWC+51.51	-	-	＜0.20
		Ra=2.33×SWC−25.29	172	<0.001	＜0.20

Table S4 Linear regression equations between soil respiration (Rs), heterotrophic respiration (Rh) and autotrophic respiration (Ra) and soil temperature and soil water content.

Treatment		Equation	N	*p*	R^2	Partial R^2	
						ST	SWC
		Rs=91.07ST−1943.50	172	<0.001	0.74	-	-
	NF	Rh=37.55ST+27.26SWC−1072.99	172	<0.001	0.99	0.53	0.47
		Ra=45.68ST+48.70SWC−460.72	172	<0.001	0.8	0.35	0.58
		Rs=99.18ST−16.66SWC−1353.43	172	<0.001	0.64	0.51	0.38
Fencing	STF	Rh=70.77ST−10.35SWC−1080.77	172	<0.001	0.99	0.68	0.32
		-	-	-	-	-	-
		Rs=51.45ST−23.70SWC+76.59	172	<0.001	0.74	0.56	0.12
	LTF	Rh=12.88ST−13.26SWC+417.13	172	<0.001	0.9	0.01	0.87
		Ra=9.63ST−7.19SWC+185.57	172	<0.001	0.99	0.09	0.9
		Rs=85.04ST+12.65SWC−1930.26	172	<0.001	0.93	0.84	0.14
	NF	-	-	-	-	-	-
		-	-	-	-	-	-
Grazing		Rs=136.67ST+3361.12	172	<0.001	0.64	-	-
	STF	Rh=73.21ST−9.67SWC+2286.88	172	<0.001	0.93	0.21	0.76
		Ra=65.25ST+1607.93	172	<0.001	0.54	-	-
	LTF	Rs=102.26ST−3.57SWC+2739.15	172	<0.001	0.69	0.52	0.38

	Rh=−2.13SWC+180.50	172	<0.001	0.82	-	-
	Ra=72.13ST−3.05SWC+1935.24	172	<0.001	0.82	0.51	0.43

NF, no flooding; STF, short-term flooding; LTF, long-term flooding; ST, soil temperature; SWC, soil water content.

Table S5 Influencing factors of Rs and its components under different flooding conditions.

Factors	NF			STF			LTF		
	Rs	Rh	Ra	Rs	Rh	Ra	Rs	Rh	Ra
ST	20.32	19.95	1.04	32.42	27.99	17.72	8.53	10.6	31.36
SWC	1.52	-	6.75		15.62	18.14	0.52	7.86	0.07
EC	8.92	2.58	15.31	3.46	1.07	3.43	3.48	2.93	1.05
pH	5.45	1.19	5.09	6.31	5.19	2.2	5.29	2.94	2.32
TC	6.5	8.17	1.88	3.11	1.46	7.44	19.94	26.55	0.04
TN	-	0.45	-	0.5	0.85	1.73	4.31	4.86	0.7
SOC	-	5.62	-	1.74	3.29	-	1.78	-	2.59
NH_4^+-N	-	1.82	27.78	3.7	4	1.82	1.72	4.75	6.32
NO_3^--N	0.84	-	8.11	0.87	1.06	-	1.09	1.41	2.37
MBC	16.95	21.69	7.93	13.03	10.69	12.9	27.86	16.2	20.85
MBN	22.88	8.2	28.97	-	-		5.39	5.34	-
BGB	2.47	4.45	-	37.15	31.63	44.25	16.35	17.49	12.96
ANPP	19.19	26.83	0.65	−	−	−	3.78	−	23.01

NF, no-flooding; STF, short-term flooding; LTF, long-term flooding; Rs, soil respiration; Rh, heterotrophic respiration; Ra, autotrophic respiration; ST, soil temperature; SWC, soil water content; EC, electrical conductivity; TC, total carbon; TN, total nitrogen; SOC, soil organic; NH_4^+-N, ammonium nitrogen; NO_3^--N, nitrate nitrogen; MBC, microbial biomass carbon; MBN, microbial biomass nitrogen; BGB, belowground biomass; ANPP, aboveground net primary production.
